# Enhancing surgical planning of distal splenopancreatectomy through 3D printed models: a case report

**DOI:** 10.1093/jscr/rjad528

**Published:** 2023-09-18

**Authors:** Stefan Arsenkov, Ognen Plavevski, Andrej Nikolovski, Ljuben Arsenkov, Arben Shurlani, Valon Saliu

**Affiliations:** Department of Abdominal Surgery, University Surgery Hospital “St. Naum Ohridski”, 1000 Skopje, North Macedonia; Independent consultant; Department of Abdominal Surgery, University Surgery Hospital “St. Naum Ohridski”, 1000 Skopje, North Macedonia; Department of Abdominal Surgery, University Surgery Hospital “St. Naum Ohridski”, 1000 Skopje, North Macedonia; Department of Abdominal Surgery, University Surgery Hospital “St. Naum Ohridski”, 1000 Skopje, North Macedonia; Department of Abdominal Surgery, University Surgery Hospital “St. Naum Ohridski”, 1000 Skopje, North Macedonia

**Keywords:** 3D printing, additive manufacturing, distal splenopancreatectomy, pancreatic resection, pancreas, surgical planning

## Abstract

The complex anatomy of the peripancreatic region was a challenge to many surgeons in the past. Up until recently, the only way to prepare and plan a surgery was through the use of traditional 2D images, obtained via computed tomography or magnetic resonance imaging. Recently, the advantages in the field of 3D printing (also called additive manufacturing, or rapid prototyping) allowed the creation of replicas of the patient’s anatomy which is to be used for preoperative planning and visual reference. We present the case of a 46-y.o. patient with a distal pancreatic lesion requiring a distal splenopancreatectomy, who benefited from the use of 3D printing technology. No intraoperative or postoperative complications were encountered, while the created model was used to plan and perform the needed resection.

## Introduction

When it comes to performing complex surgical procedures like a distal splenopancreatectomy, surgical planning plays a vital role. Traditionally, surgeons have relied on preoperative imaging modalities, such as computed tomography (CT) and magnetic resonance imaging (MRI), to visualize anatomical structures and plan resections and approaches.

Over the past few years, 3D printing technology has experienced significant advancements, making it more accessible and cost-effective. We present the case of a patient diagnosed with a distal pancreatic mass requiring surgical intervention. A 1:1 replica of the patient’s unique anatomy was manufactured using a 3D printer, and it was used for preoperative planning and intraoperative visual reference.

## Case report

**Figure 1:**
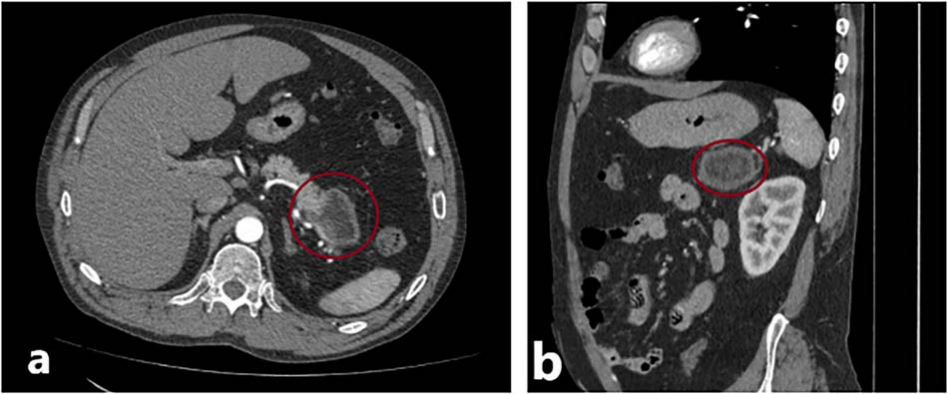
(a) and (b) Axial and sagittal CT images showing a distal pancreatic tumor.

The patient, a 46-y.o. male was referred to our hospital with an already diagnosed pancreatic lesion with a diameter of ~5 cm. The patient contacted their general practitioner (GP) because of nonspecific upper-abdominal symptoms, such as vague pain in the upper abdomen, bloating, and occasional vomiting. The GP ordered an abdominal ultrasound which showed a pancreatic mass, leading to a CT scan being done. The patient’s lab results were within normal values all along. Based on the CT scan findings (Fig. 1), a decision to operate was made. A distal splenopancreatectomy being a complex procedure, a decision to try and print a 3D model of the patient’s pancreas, spleen, and surrounding vessels was reached.

**Figure 2:**
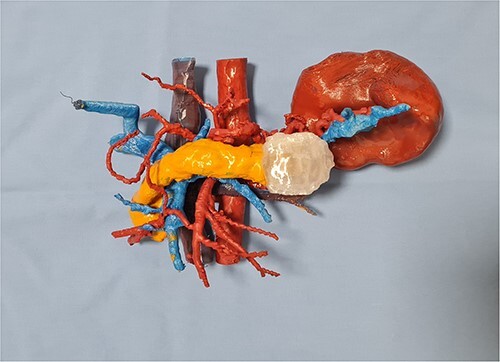
The 3D printed replica of the patient’s anatomy.

In our case report, we employed a combination of CT scanning and open-source software, which enabled the segmentation and reconstruction of the patient’s organs from the CT images. By precisely delineating the boundaries of the organs of interest, we created a digital representation that faithfully replicated the patient’s unique anatomy. Subsequently, the digital model was transformed into a physical replica. The resulting 3D printed model provided the surgical team with a tangible and realistic representation of the patient’s pancreas, spleen, aorta, vena cava, and portal venous system (Fig. 2).

**Figure 3:**
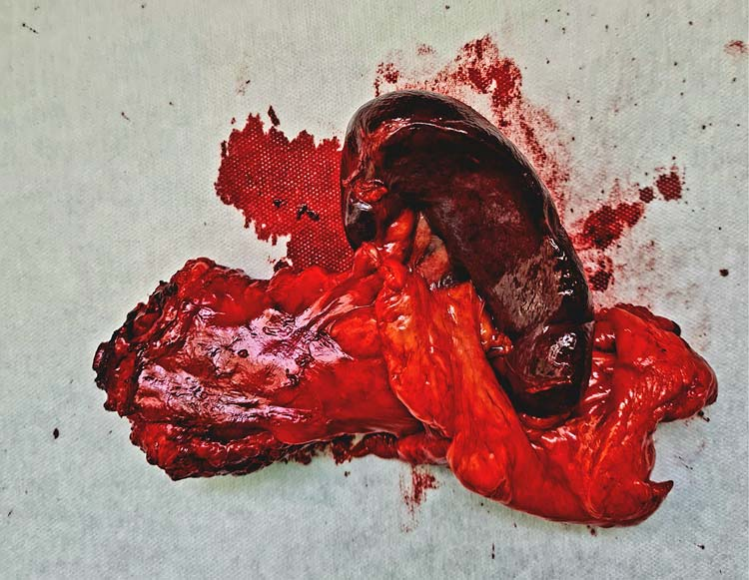
The resected specimen—distal pancreas and spleen with lesion in the middle of the specimen.

Once the 3D printed model was manufactured, it was presented to the surgical team with the immediate consensus being that the tangible object significantly enhanced their understanding of the patient’s unique anatomy, surpassing the comprehension derived from both the 2D CT scan images and the pre-printing 3D renderings. The ability to hold and manipulate the model provided a tactile experience that facilitated a deeper appreciation of the spatial relationships between the organs involved in the distal splenopancreatectomy procedure. The precise and accurate replication of the patient’s anatomy allowed for the identification of critical factors, such as optimal resection margins and planning the approach to the blood vessels in the vicinity. The inferior mesenteric vein was used as a landmark, and its relation to the portal and splenic vein was studied in depth. This landmark was used intraoperatively, when after the adequate organ mobilization, the inferior mesenteric vein was obtained in view. The resected specimen, spleen, and tail of pancreas, is shown in Fig. 3. It is worth noticing that the lesion is not as easily noticeable as it is on the 3D printed model.

In the postoperative period, the patient’s recovery progressed smoothly and uneventfully. No complications were encountered and on postoperative day 5, the patient was discharged from the hospital.

## Discussion

The utilization of 3D printing in orthopedic and maxillofacial surgery has been particularly prominent. However, the application of 3D printing in soft-tissue surgery, such as abdominal surgery, has been less widespread. This discrepancy can be attributed to the complex nature of soft-tissue structures, the challenges associated with replicating their intricate features [1], and the limited availability of suitable biocompatible materials for 3D printing. Although the integration of 3D printing technology into soft-tissue surgeries, like abdominal procedures, has been documented and the benefits noted [2], 3D printing remains an area of ongoing research and exploration. While the number of published articles is rising in recent years, most of the articles are focused on liver and kidney models. [3]

The necessity to enhance the process of surgical planning in pancreatic surgery has been the mother of several attempts at devising an improved approach. In 2007, Brennan *et al.* [4] worked on creating a virtual Whipple procedure. They also noted the highly variable anatomy of the pancreatic region. Although the advantages of using such models were noted almost a decade ago, especially when it comes to education of surgeons [5], the usage and reports have yet to be widely implemented, currently being limited to small series, such as those published by Schwaiger [6] and Kontovounisios *et al.* in 2019 [7].

Our impression was that the anatomical features of the healthy tissues and tumor were visualized to a greater detail on the 3D model compared to the 2D images, and compared to real-life structures, corroborating the findings in other studies. Izatt *et al.* [8] found in 2007 that in 65% of cases the structures were better visualized on a 3D printed model, and exclusively visible on the 3D printed model in 11% of cases. In our case, by simulating the surgical procedure on the model, the surgical team could anticipate potential challenges and determine the most suitable techniques to safely navigate and preserve the delicate vascular structures.

## Conclusion

In summary, the utilization of the 3D printed model provided the surgical team with an enhanced understanding of the patient’s anatomy, surpassing the limitations of traditional 2D imaging and pre-printing 3D renderings. Its tangible nature facilitated an immersive experience that aided in formulating a comprehensive preoperative plan, specifically concerning resection margins and the approach to vital blood vessels. The notable reduction in the duration of the surgical procedure, might be seen as an additional significant benefit derived from the utilization of the 3D printed model. In this case report, the distal splenopancreatectomy operation was successfully completed within a remarkably shorter timeframe of ~2.5 h. This duration stands in stark contrast to the median duration reported in existing studies for similar operations, which often extend beyond this timeframe [9, 10]. The integration of 3D printing technology into surgical planning for distal splenopancreatectomy showcased its immense potential to optimize surgical strategies, enhance patient outcomes, and advance the field of abdominal surgery.

## Data Availability

The data underlying this article are available in the article and in its online supplementary material.
